# Multiple cell-death patterns predict the prognosis and drug sensitivity of melanoma patients

**DOI:** 10.3389/fphar.2024.1295687

**Published:** 2024-10-08

**Authors:** Zewei Chen, Ruopeng Zhang, Zhoukai Zhao, Baiwei Zhao, Feiyang Zhang, Guoming Chen, Xiaojiang Chen, Chengzhi Wei, Jun Lin, Feizhi Lin, Ziqi Zheng, Kaiming Jiang, Runcong Nie, Yingbo Chen

**Affiliations:** ^1^ State Key Laboratory of Oncology in South China, Collaborative Innovation Center for Cancer Medicine, Sun Yat-Sen University Cancer Center, Guangzhou, China; ^2^ Department of Gastric Surgery and Melanoma Surgical Section, Sun Yat‐Sen University Cancer Center, Guangzhou, China

**Keywords:** melanoma, postoperative prediction model, programmed cell death, drug sensitivity, cell death index

## Abstract

**Background:**

Melanoma, a malignant tumor of the skin, presents challenges in its treatment process involving modalities such as surgery, chemotherapy, and targeted therapy. However, there is a need for an ideal model to assess prognosis and drug sensitivity. Programmed cell death (PCD) modes play a crucial role in tumor progression and has the potential to serve as prognostic and drug sensitivity indicators for melanoma.

**Methods:**

We analyzed 13 PCD modes including apoptosis, necroptosis, ferroptosis, pyroptosis, netotic cell death, entotic cell death, lysosome-dependent cell death, parthanatos, autophagy-dependent cell death, oxeiptosis, disulfidptosis, and alkaliptosis. These modes were used to construct a model that incorporated genes related to these 13 PCD modes to establish a cell death index (CDI) to conduct prognosis analysis. Transcriptomic, genomic, and clinical data were collected from cohorts including TCGA-SKCM, GSE19234, and GSE65904 to validate this model.

**Results:**

A CDI consisting of ten gene signatures was established using machine learning algorithms and divided into two groups based on CDI values. The high CDI group exhibited relatively lower numbers of immune-infiltrating cells and showed resistance to commonly used drugs such as docetaxel and axitinib. Our validation results demonstrated good discrimination in PCA analysis between CDI groups, and melanoma patients with higher CDI values had worse postoperative prognoses (all p < 0.01).

**Conclusion:**

The CDI model, incorporating multiple PCD modes, accurately predicts the clinical prognosis and drug sensitivity of melanoma patients.

## 1 Introduction

Melanoma is the most lethal type of skin cancer, originating from the malignant transformation of melanocytes. Globally, melanoma accounts for approximately 1.7% of all newly diagnosed primary malignant cancers, and deaths from melanoma constitute around 0.7% of all cancer mortality (Schadendorf et al., 2018). The incidence and mortality rates of melanoma vary between countries (Schadendorf et al., 2018). According to estimates from the American Cancer Society, the number of new cases of melanoma is projected to reach 97,610, with 7,990 deaths, by 2023 (Siegel et al., 2023). Surgery currently remains the primary treatment modality for early-stage melanoma (Zheng et al., 2020). With advances in the understanding of the pathogenesis of melanoma, it has been recognized that gene mutations play a crucial role in its development. In particular, the wide application of targeted therapy and immunotherapy has substantially improved the 5-year survival rate of patients with advanced melanoma from <10% to around 30% (Guo et al., 2021). Therefore, in order to improve the prognosis of melanoma, there is an urgent need to explore new targets and establish effective models—necessary prerequisites for making targeted therapies more feasible (Mahdavi et al., 2021; Khajuria and Agha, 2013).

Cell death can be categorized into accidental cell death (ACD) and programmed cell death (PCD) depending on the triggering mechanisms. PCD encompasses various forms of cell death, including apoptosis, necroptosis, ferroptosis, pyroptosis, netotic cell death, entotic cell death, lysosome-dependent cell death, parthanatos, autophagy-dependent cell death, oxeiptosis, disulfidptosis, and alkaliptosis, which are regulated by complex processes (Tang et al., 2019). Netotic cell death is a neutrophil-induced form of cell death that is characterized by the release of DNA that forms extracellular web-like structures (Brinkmann et al., 2004). Alkaliptosis is a newly recognized form of cell death, marked by metabolic dysfunction under alkaline conditions (Song et al., 2018). PCD serves as a natural defense mechanism against the survival and dissemination of cancer cells. However, cancer cells evade PCD through different strategies, such as acquiring genetic mutations or epigenetic modifications in key PCD pathway regulators (Su et al., 2015). The relationship between PCD and melanoma has not been fully elucidated, and there is limited research on the specific functional aspects of PCD in melanoma. Although previous studies have partially investigated the relationship between melanoma and programmed cell death, such as apoptosis and ferroptosis (Liu C. et al., 2022; Hussein et al., 2003), there is still little analysis on how melanoma integrates multiple modes of cell death. Therefore, this study aims to introduce a novel indicator, the cell death index (CDI), to improve prognostic prediction in melanoma. Overall, our research highlights the heterogeneity among melanoma patients and evaluates the clinical prognosis of this disease. These findings may assist melanoma patients to make informed decisions about appropriate treatment strategies.

## 2 Methods

### 2.1 Data collection

PCD-related genes include apoptosis, necroptosis, ferroptosis, pyroptosis, netotic cell death, entotic cell death, lysosome-dependent cell death, parthanatos, autophagy-dependent cell death, oxeiptosis, disulfidptosis, and alkaliptosis. These genes are collected from GSEA gene sets, KEGG, review articles, and manual collation (Tang et al., 2019; Liu and Tang, 2023). The final gene list represents a comprehensive compilation of regulatory genes spanning all 13 PCD patterns. A total of 580 apoptosis genes, 5 alkaliptosis genes, 292 autophagy genes, 14 cuproptosis genes, 14 disulfidptosis genes, 7 entotic cell death genes, 78 ferroptosis genes, 176 lysosome-dependent cell death genes, 51 necroptosis genes, 7 netotic cell death genes, 1 oxeiptosis gene, 7 parthanatos genes, and 36 pyroptosis genes were included in this study. In total, 1,268 concatenated genes associated with PCD patterns were incorporated for analysis.

To conduct the analysis, we obtained RNA-seq transcripts per million (TPM) data from a cohort comprising 469 melanoma patients and 557 normal samples by accessing the TCGA and GTEx databases through the University of California Santa Cruz (UCSC) database; the data had undergone log transformation. Ensuring accurate gene annotation, we utilized the AnnoProbe R package to convert ensemble IDs to gene symbols, and clinical features were acquired from the official TCGA website. Additionally, we retrieved log-transformed chip-seq data and corresponding clinical characteristics of a separate cohort containing 258 melanoma patients from the Gene Expression Omnibus (GEO) database (ID: GSE19234, GSE65904) for external validation purposes, with probe mapping facilitated using the AnnoProbe R.

### 2.2 Expression, annotation, and genetic mutation information about PCD-related genes

In the TCGA-SKCM and GTEx cohorts, we prepared raw transcriptome data from 469 melanoma patients and 557 normal tissues. The “limma” package was subsequently used to identify differentially expressed genes (DEGs) (Ritchie et al., 2015), with the criteria of adjusted p < 0.05 and |log2FC| > 1. The visualization of DEGs was performed using the “pheatmap” and “ggplot “packages. We utilized the “maftools” package to explore genetic mutation information among melanoma patients (Mayakonda et al., 2018).

### 2.3 Development of the PCD-related gene signature

We used univariate Cox regression analysis to evaluate the impact of these genes on the survival status of melanoma patients. To ensure the accuracy of the model, we adjusted the significance threshold to 0.05. Furthermore, we employed the LASSO Cox regression method to narrow down the candidate gene set and construct the optimal gene signature. We selected the “lambda.min” value using the “glmnet” R package (Friedman et al., 2010).

The CDI for each patient was calculated using the formula $CDI=\sum_{i=1}^{10}\beta i*Ei$ . Β i represents the risk coefficient associated with each gene, and E i denotes the corresponding gene’s expression level. For a more intuitive visualization, we divided melanoma patients into low- and high-CDI groups based on the median CDI value. Using the patient’s clinical information and CDI values, we created a clustered heatmap using the “pheatmap” package in R. Additionally, we examined the relationship between CDI grouping and the clinical information of melanoma patients using Wilcox, t-, and ANOVA tests.

### 2.4 Functional enrichment

We utilized the “clusterProfiler” R package to identify possible biological pathways based on the DEGs and the genes identified through lasso regression (Wu et al., 2021). Furthermore, we employed the GSVA and GSEABase packages in R to analyze the differential biological functions between the high- and low-CDI groups (Wu et al., 2021).

### 2.5 Tumor microenvironment analysis and drug sensitivities

The association between CDI and immunomodulators, as well as immune cells, was analyzed. The single-sample gene set enrichment analysis (ssGSEA) and CIBERSORT deconvolution algorithm were employed to analyze the cellular composition of the tissues based on gene expression profiles (Newman et al., 2015). Additionally, drug sensitivities were predicted using “oncoPredict” (Maeser et al., 2021).

### 2.6 Internal training and external validation of the gene signature prediction model

External validation was conducted using the GSE19234 and GSE65904 datasets. Scatter plots were generated using the “ggplot” package depicting the relationship between CDI grouping and survival outcomes. Principal component analysis (PCA) was performed using the “stats” package. Additionally, Kaplan–Meier analysis, examining the correlation between OS time and CDI, was conducted using the “survival” and “survminer” packages. Furthermore, receiver operating characteristic (ROC) analysis of CDI was performed using the “timeROC” R package (Blanche et al., 2013).

### 2.7 Establishment and assessment of the nomogram survival model

We collected clinical information of melanoma patients from the TCGA database, including age, gender, T stage, N stage, and stage grouping. We performed univariate and multivariate Cox regression analyses using the clinical information and CDI values. The patients were then stratified into stage IV and non-stage IV groups, and we developed a prognostic nomogram and calibration curve incorporating age, gender, T stage, N stage, and CDI values using the “rms” and “survival” packages in R (Harrell Jr, 2023). ROC analysis of the nomogram was performed using the R package “timeROC” (Blanche et al., 2013). Additionally, we compared the nomogram models with and without CDI using pROC and a Wilcoxon rank-sum test (Su et al., 2015). Furthermore, based on the outcomes derived from the nomogram analysis including and excluding CDI, the participants were stratified into high- and low-risk cohorts using the mean score computed from their respective datasets. Consequently, Kaplan–Meier survival curves were constructed for each cohort. It is important to note that for the external validation of the nomogram survival model, we did not include T and N variables due to limited data availability for melanoma.

### 2.8 Statistical analysis

All statistical analyses were performed using R version 4.2.1 Two-group comparisons were conducted using t-tests or Wilcoxon tests. Overall comparisons were done using ANOVA. Survival curves were analyzed using the log-rank test and described using Kaplan–Meier plots. p < 0.05 was considered statistically significant.

## 3 Results

### 3.1 Study workflow

**FIGURE 1:**
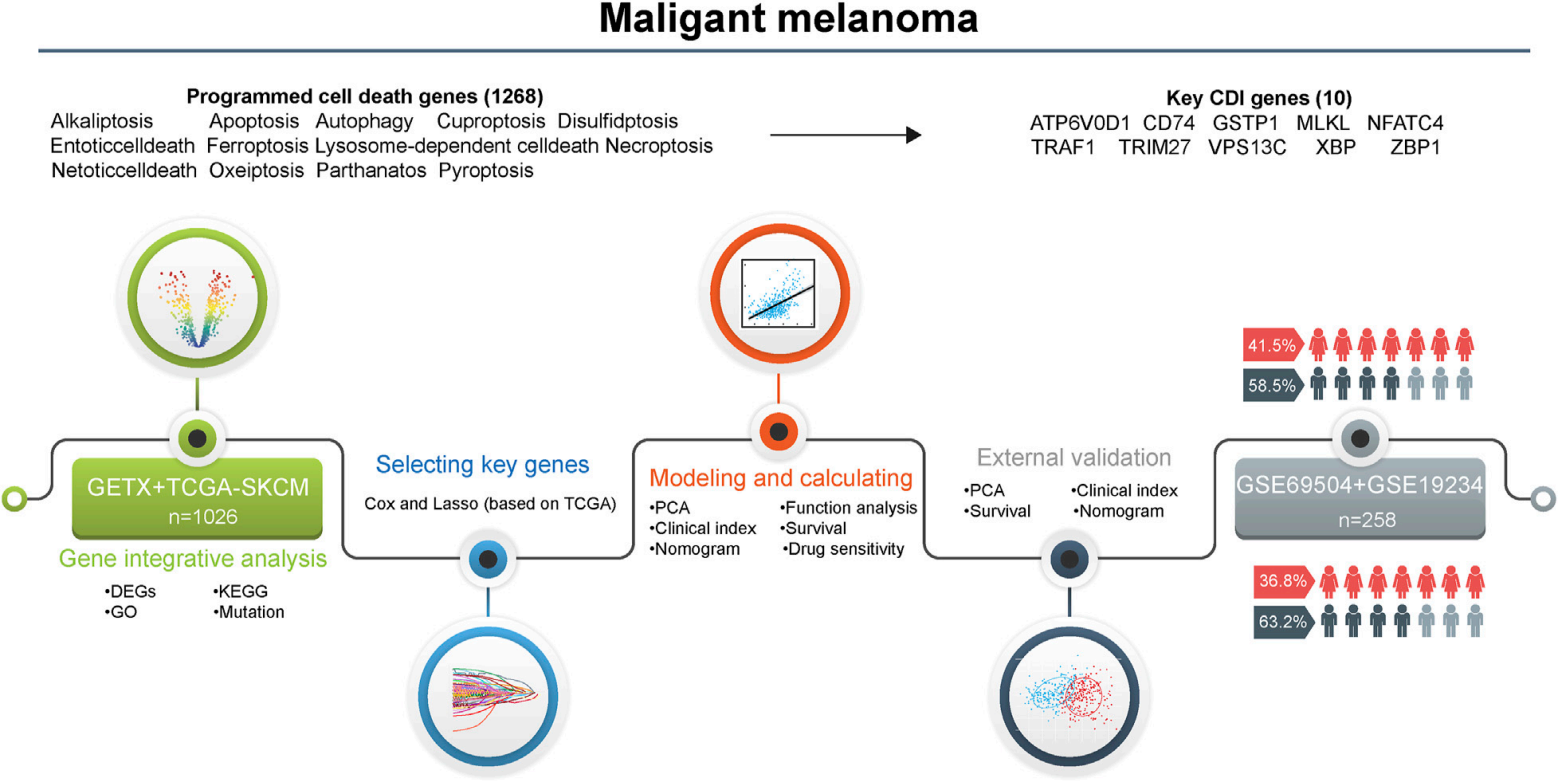
Flowchart for the analysis of cell death patterns in patients with melanoma. Abbreviations: DEGs, differentially expressed genes; KEGG, Kyoto encyclopedia of genes and genomes; GO, gene ontology; PCA, principal component analysis; CDI, cell death indicator.

We selected 1,267 genes related to programmed cell death (PCD) and identified 1,026 patients from the TCGA and GTEx databases, 44 from GSE19234, and 214 patients from GSE65904 for inclusion in the analysis. Figure 1 illustrates the workflow employed in this study.

### 3.2 Differentially expressed genes (DEGs) related to programmed cell death in the TCGA cohort and GTEx cohort

**FIGURE 2:**
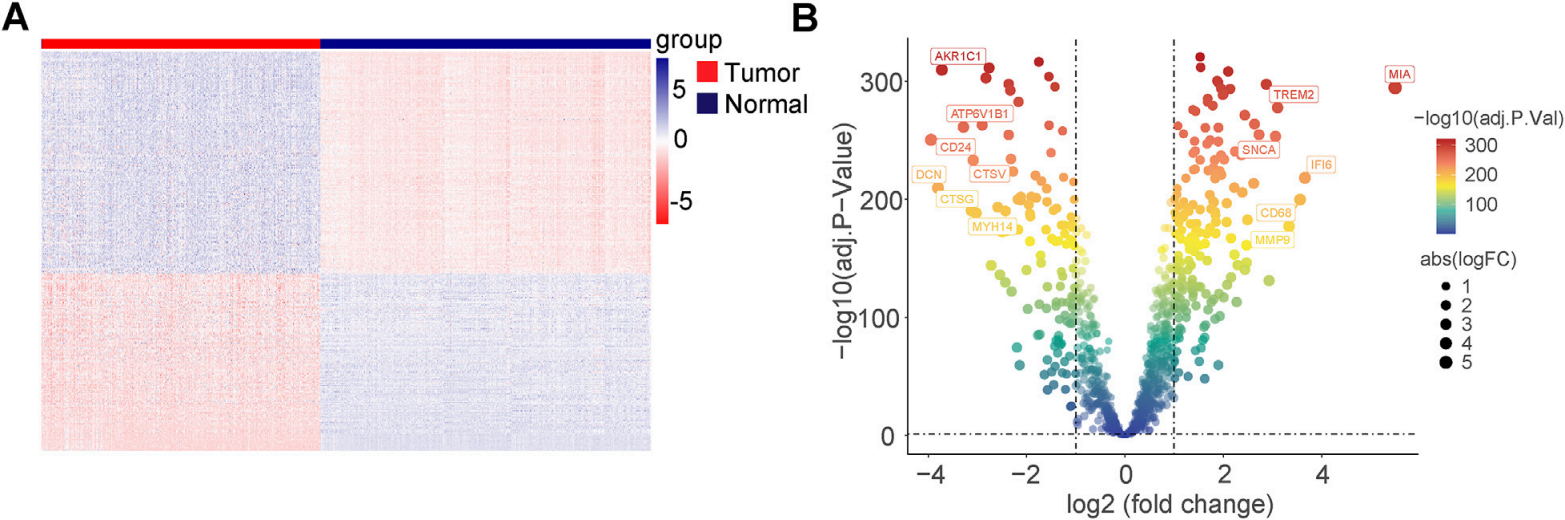
Differentially expressed genes (DEGs) related to programmed cell death in TCGA cohort and GTEx cohort. **(A)** Heatmap of the PCD-related DEGs between malignant melanoma and normal tissues. **(B)** Volcano plot of the PCD-related DEGs between malignant melanoma and normal tissues (two vertical lines indicate gene expression fold change >1 and <−1, respectively, and the horizontal line indicates the P-value of 0.05; points with labels are obvious DEGs which |log2FC| > 3).

In the TCGA and GETX cohorts, 335 DEGs were identified (all adjusted; p < 0.05, and |log2FC| > 1), of which 149 were upregulated and 186 were downregulated in the melanoma group. The normalized RNA levels of the DEGs are displayed as heatmaps in Figure 2A, while the volcano plot of the DEGs is presented in Figure 2B (two vertical lines indicate gene expression fold change >1 and <−1, respectively, and the horizontal line indicates the P value of 0.05. Points with labels are obvious DEGs which |log2FC| > 3). In addition, gene ontology (GO) and Kyoto Encyclopedia of Genes and Genomes (KEGG) enrichment analysis indicated that these DEGs participated in multiple biological pathways, such as lysosome, intrinsic apoptotic signaling pathway, and autophagosome (Supplementary Figures 1A, B). Furthermore, the variation in PCD-related genes was evaluated in melanoma patients from the TCGA cohort. Results showed that about 327/456 (71.71%) of melanoma patients had mutations. Missense mutation is the main mutation type, accounting for about 90% of all mutations. The top-20 mutations of PCD-related genes were displayed, with DCC possessing the highest mutation frequency (22%), with 19 others ranging from 18% to 10% (Supplementary Figures 1C, D).

### 3.3 Construction of a prognostic gene signature for malignant melanoma patients

**FIGURE 3:**
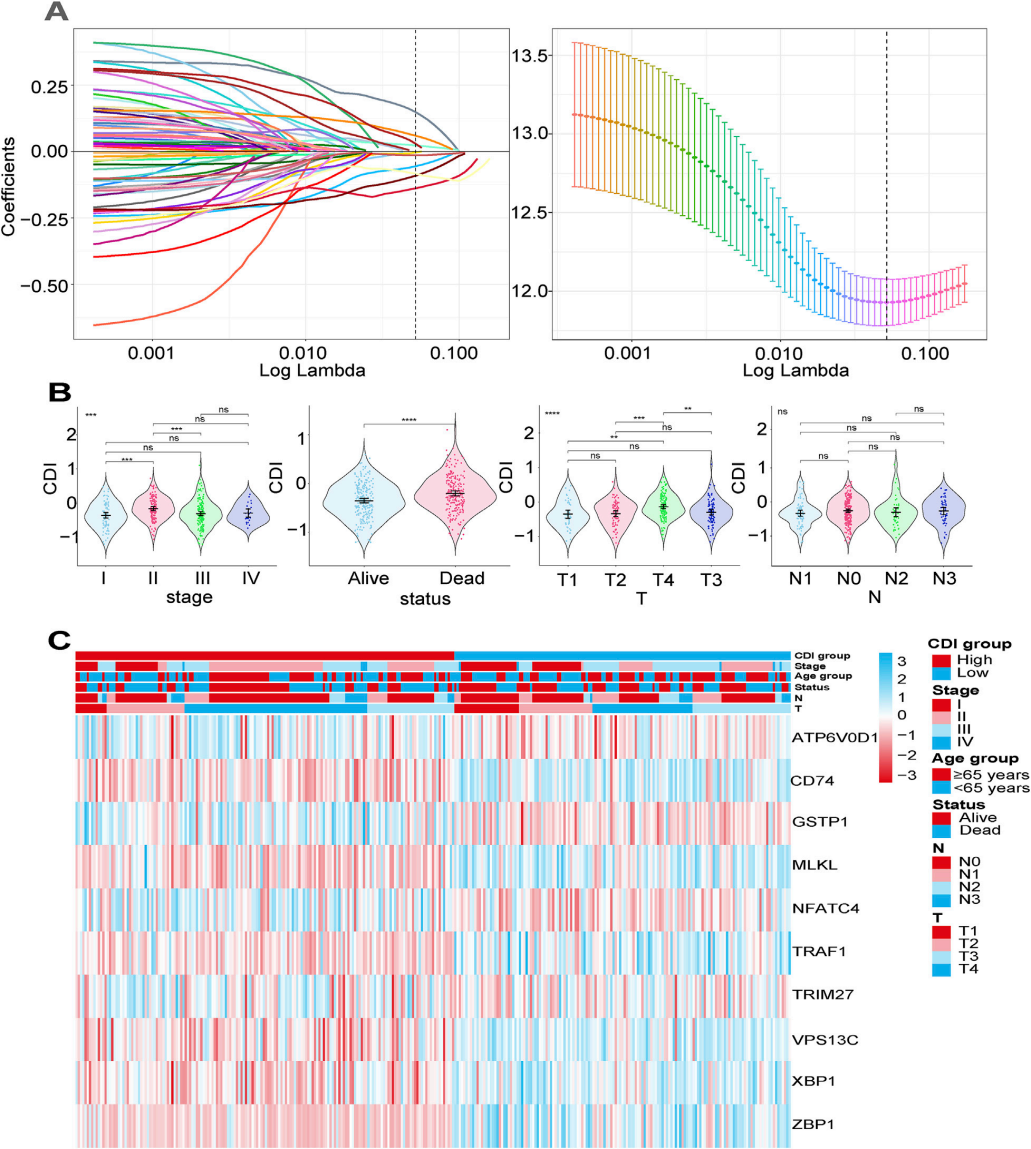
Construction of a prognostic gene signature for malignant melanoma patients. **(A)** Selection of the 10 model genes by lasso regression, machine learning method, and cross-validation of the constructed signature. **(B)** Violin plots of the relationship between CDI and clinical information on different melanoma patients. ****p < 0.0001; **p < 0.01; *p < 0.05; ns, not significant. **(C)** Heatmap of 10 model genes and clinical features.

We collected and analyzed survival information of melanoma patients using univariate Cox regression analysis to screen for genes associated with survival. In TCGA, 357 genes, and in GSE19234, 181 genes, met the cutoff criteria of p < 0.05, and the intersection of the two screenings contained 67 genes. Through lasso Cox regression analysis, we constructed a signature consisting of ten genes (ATP6V0D1, CD74, GSTP1, MLKL, NFATC4, TRAF1, TRIM27, VPS13C, XBP1, and ZBP1), with five from apoptosis, three from autophagy, and two genes from necroptosis (Figure 3A), which not only play a crucial role in the pathogenesis and the development of melanoma but also serve as a potential factor in reshaping the immune microenvironment, mediating metastasis, and influencing drug resistance. This highlights the significant impact of multiple PCD pathways in melanoma cell biology and their implications in modulating a tumor’s biological behaviors (Zou et al., 2022). We employed Kaplan–Meier analysis (log-rank test) to evaluate the impact of these ten genes on overall survival (OS) time. Our findings demonstrated that higher expression levels of CD74, MLKL, TRAF1, VPS13C, XBP1, and ZBP1 were associated with improved survival outcomes in patients, while the higher expression levels of ATP6V0D1, GSTP1, NFATC4, and TRIM27 were correlated with poorer survival outcomes (all p < 0.05) (Supplementary Figure 2). T-tests were performed to compare the expression levels of these genes between melanoma tissues and normal samples. Of these, ATP6V0D1, CD74, TRIM27, TRAF1, XBP1, and ZBP1 showed higher expression in tumor tissues, while MLKL, VPS13C, and NFATC4 showed lower expression in tumor tissues (all p < 0.05), and the expression of GSTP1 did not show a significant difference between melanoma tissues and normal samples (Supplementary Figure 3). We derived the CDI for each patient using a formula based on the expression levels of the 10 genes. Based on the calculated median CDI, we divided the 451 melanoma patients in the TCGA cohort into high and low CDI groups as the training dataset. CDI was significantly associated with clinical characteristics including survival status, T stage, and clinical stage (p < 0.05) but not N stage (Figure 3B). A heatmap illustrating the expression profiles of the ten model genes and their associations with clinical characteristics is provided. The figure demonstrates that the elevated expression of certain genes is strongly correlated with poor prognosis, such as decreased survival rates or more advanced tumor stages. For instance, ATP6V0D1 shows significant upregulation in patients with advanced tumor stages, while TRIM27 is notably associated with worse overall survival outcomes (Figure 3C).

### 3.4 Internal training and external validation of the gene signature prediction model

**FIGURE 4:**
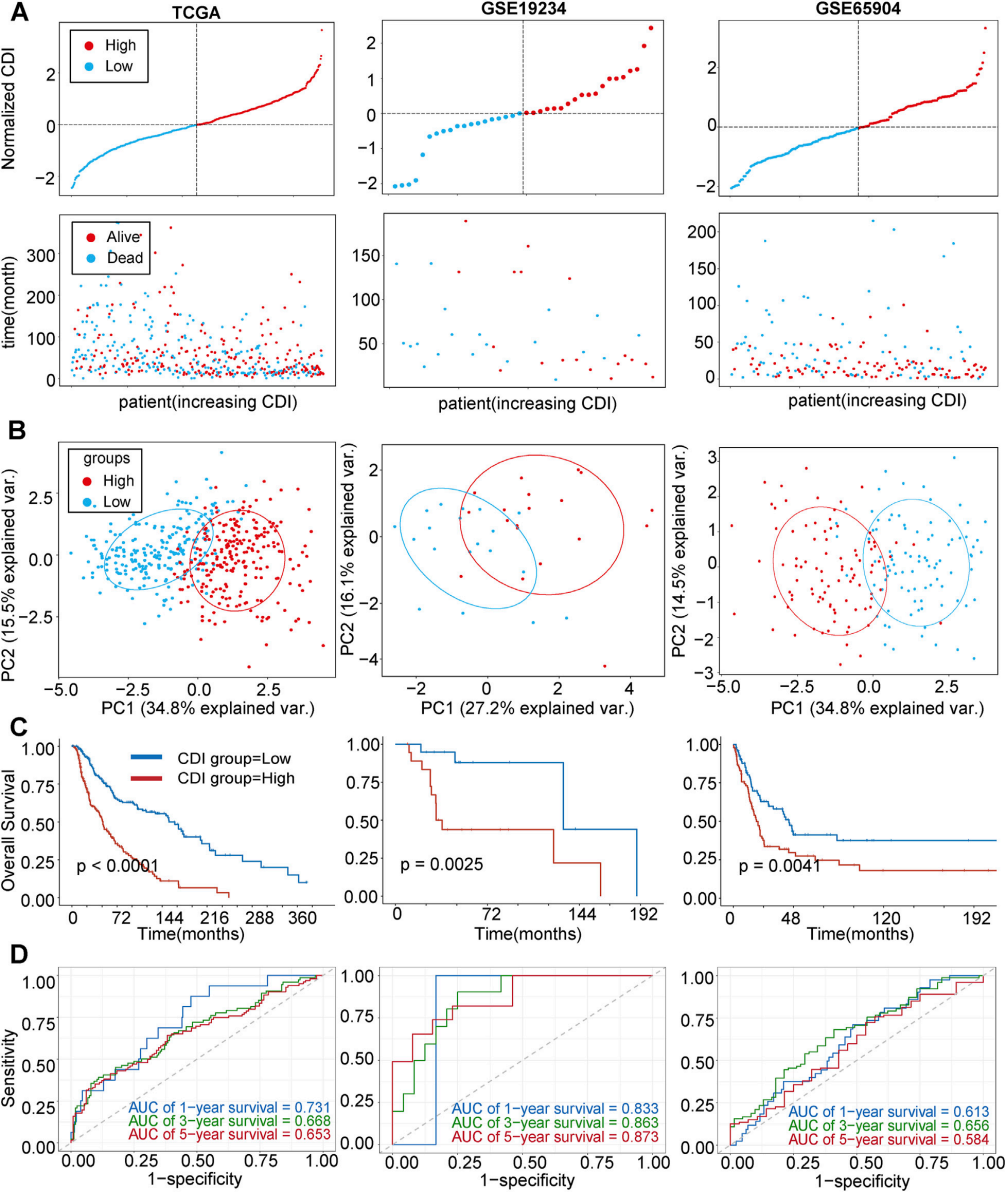
Internal training and external validation of the gene signature prediction model. **(A)** Distribution of adjusted CDI according to survival status and time in TCGA, GSE19234, GSE65904 cohorts. **(B)** Principal component analysis (PCA) plot based on the CDI in TCGA, GSE19234, and GSE65904 cohorts. **(C)** Overall survival in the low- and high-CDI group patients in TCGA, GSE19234, and GSE65904 cohorts. **(D)** Receiver operating characteristic (ROC) analysis of CDI in TCGA, GSE19234, and GSE65904 cohorts. Abbreviations: AUC, area under the curve.

In the following analysis, we examined the differences in OS between melanoma patients with varying CDI values. The results revealed that patients with high CDI levels exhibited a lower OS rate than those with low CDI (Figure 4A), with their 5-year survival rate being 22.2% versus 48.2%. Utilizing principal component analysis (PCA), we observed distinct separation based on CDI values. Samples from the high CDI group clustered more closely in the PCA plot, further supporting the consistency of gene expression and clinical characteristics within this group (Figure 4B). Furthermore, there was a significant disparity in OS times between the two groups, with low-CDI patients demonstrating increased survival rates (HR = 0.36, 95% CI: 0.27–0.48, p < 0.001, Figure 4C). To validate our findings, GSE19234 and GSE65904 were used as independent cohorts, with patients divided into high and low CDI groups based on median CDI for each cohort. The results demonstrated a correlation between higher CDI and shorter survival time (Figure 4A). Additionally, PCA showed clear separation between the two groups (Figure 4B), while Kaplan–Meier analysis indicated that the low-CDI group had better OS and lower death rates (HR = 0.27, 95% CI: 0.10–0.71, p = 0.003 and HR = 0.56, 95% CI: 0.38–0.84, p = 0.004, Figure 4C). ROC analysis of CDI values in three independent cohorts supported our findings, indicating the significant accuracy of CDI values in predicting the 1-, 3-, and 5-year OS of melanoma patients (Figure 4D). The AUC values for 1-, 3-, and 5-year survival in the training set were 0.731, 0.668, and 0.653, and the validation set showed AUCs ranging from 0.584 to 0.873.

### 3.5 Function enrichment and immune analysis between two CDI groups in the TCGA cohort

**FIGURE 5:**
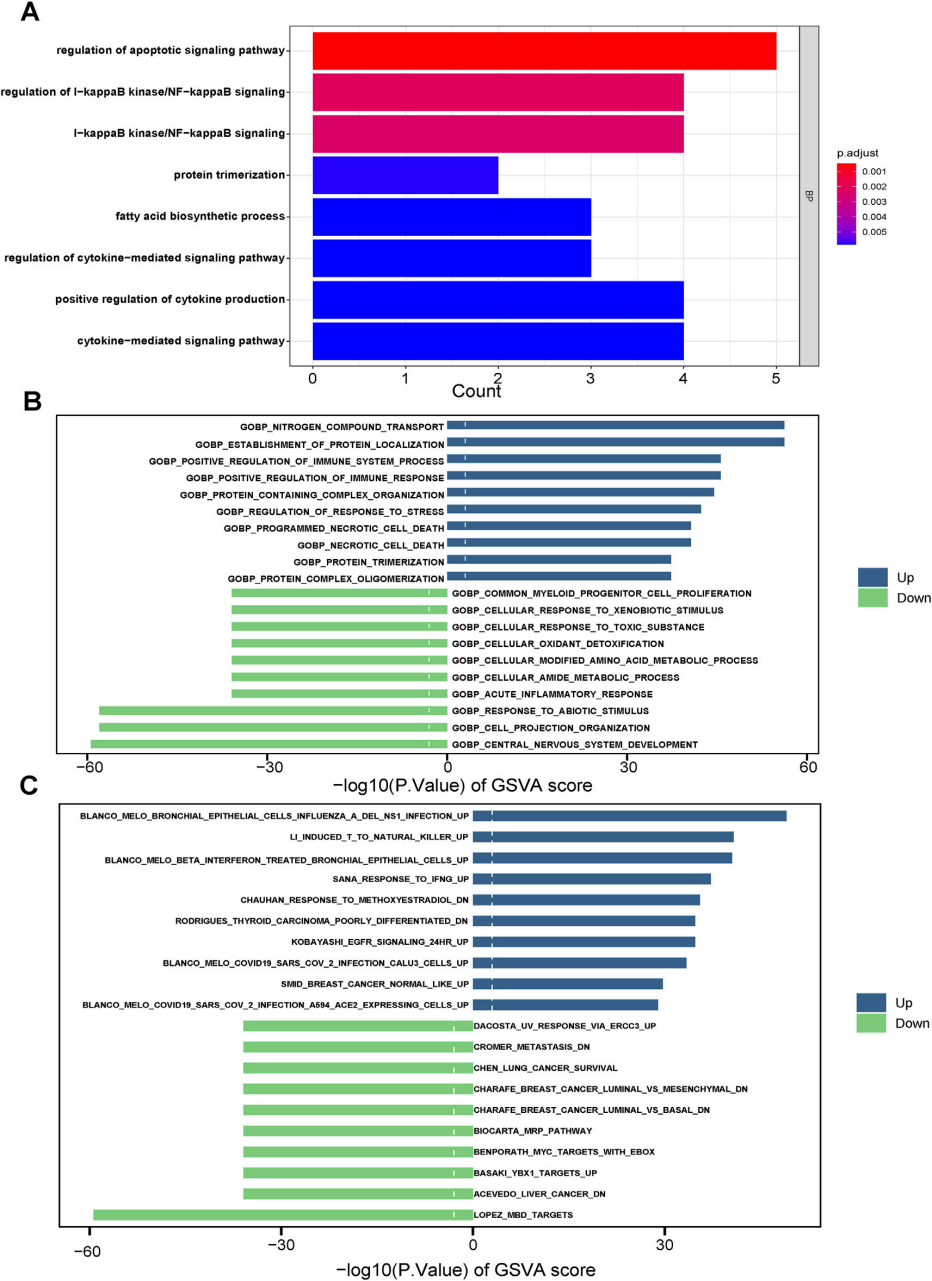
Plot of enrichment biological functions between two CDI groups in TCGA cohort. **(A)** GO enrichment analyses based on the 10 model genes. **(B, C)** GSVA between two CDI groups in TCGA cohort. Abbreviations: GVSA, gene set variation analysis.

**FIGURE 6:**
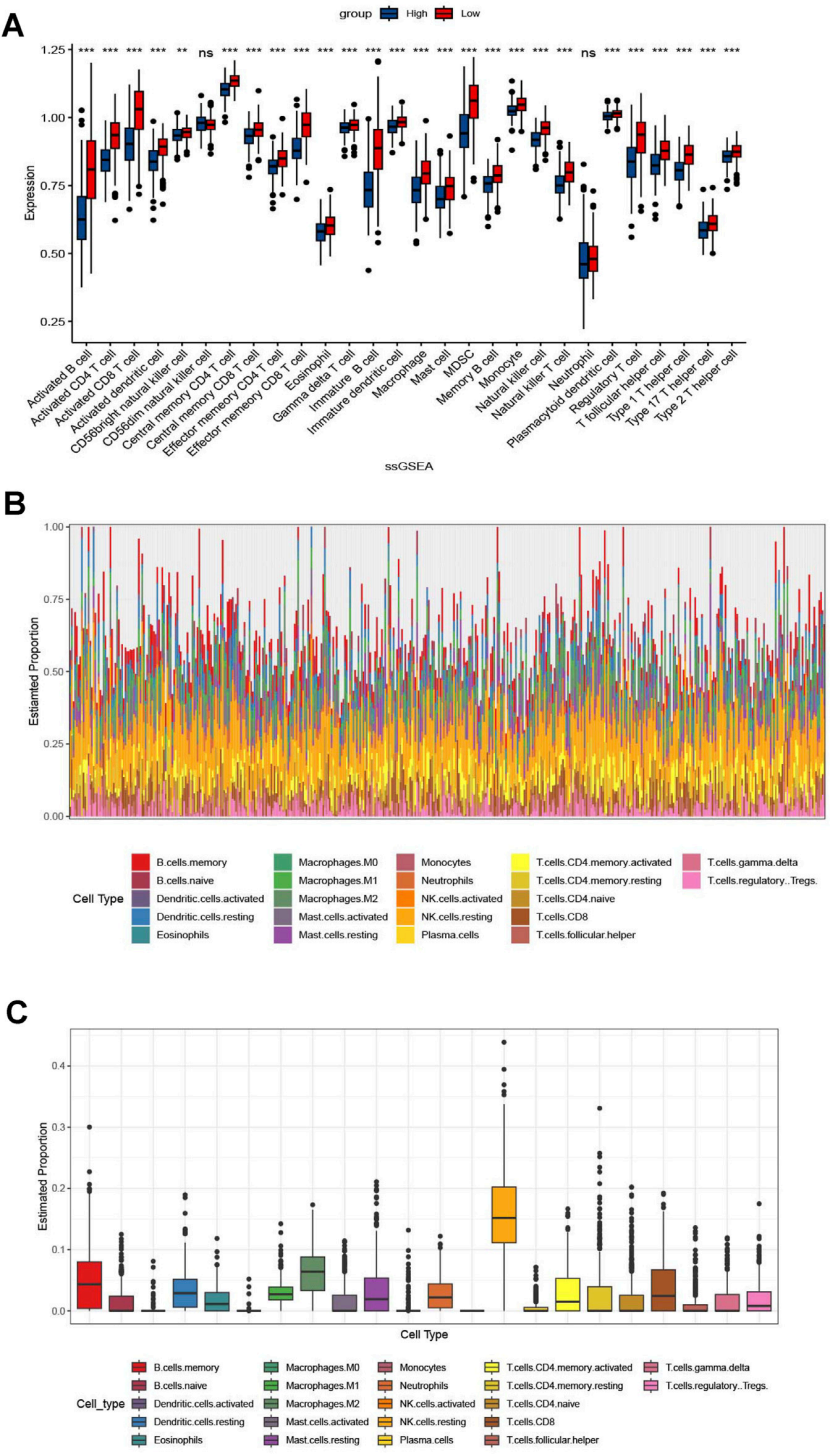
Correlation between immunomodulators and CDI values in malignant melanoma patients. **(A)** Box plot of the correlation between immunomodulators and CDI values in malignant melanoma patients. **(B, C)** Supplementary Figure based on Figure **(A)**.

KEGG analysis revealed significant enrichment of the cell apoptosis pathway (p < 0.001, Figure 5A). In GEVA analysis, we selected the top-ten upregulated and top-ten downregulated pathways (Figures 5B, C). Upregulation was observed in pathways related to nitrogen compound transport, while pathways associated with the nervous system showed downregulation. Additionally, immune analysis indicated a relatively higher presence of immune-infiltrating cells in the CDI low group, accounting for 26 out of 28 (92.8%) of the total number and proportion of immune cells in this group. Specifically, activated B cells, activated CD8 T cells, and immature B cells were significantly higher in the CDI low group (p < 0.001), while CD56dim natural killer cells and neutrophils did not show significant differences between the groups (Figure 6). The marked decrease in these critical tumor-killing cells within the CDI low expression group underscores the pivotal role of the CDI score in assessing the anti-tumor microenvironment. A lower CDI is indicative of a more suppressive tumor microenvironment, consistent with poorer survival outcomes, and aligns with the findings of previous studies (Cao et al., 2024).

### 3.6 Relationship between tumor drug sensitivity and CDI

**FIGURE 7:**
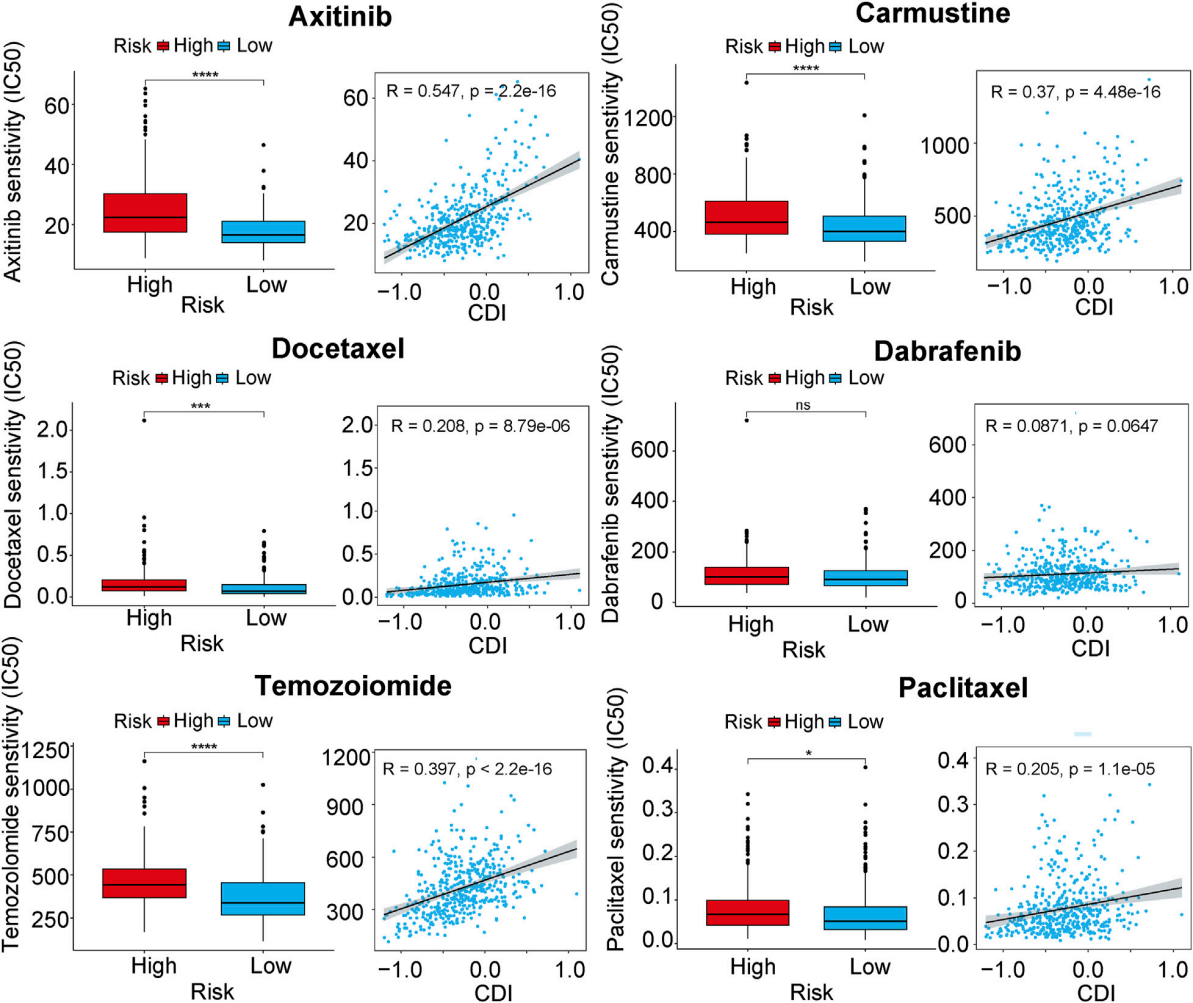
Boxplots of the comparison of IC_50_ of drugs between high- and low-CDI groups, and correlation between IC_50_ and CDI values in TCGA cohort in melanoma-related drugs. Abbreviations: IC_50_, half-maximal inhibitory concentration.

We conducted an analysis to determine whether there were differences in other important features between the two CDI groups. We calculated the half maximal inhibitory concentration (IC50) values of commonly used melanoma drugs to explore the relationship between the established model and drug sensitivity. The scatter plots and box plots of the correlation and significance between drug sensitivities and CDI for six commonly used melanoma drugs (Axtinib, Carmustine, Docetaexl, Temozolomide, Paclitaxel, Dabrafenib) are presented in Figure 7. We found that the IC50 values of these drugs were higher in the high-CDI group, which may suggest that melanoma patients with high CDI are resistant to standard chemotherapy regimens and result in poor prognosis. This finding is also consistent with previous results.

### 3.7 Establishment and assessment of the nomogram survival model

**FIGURE 8:**
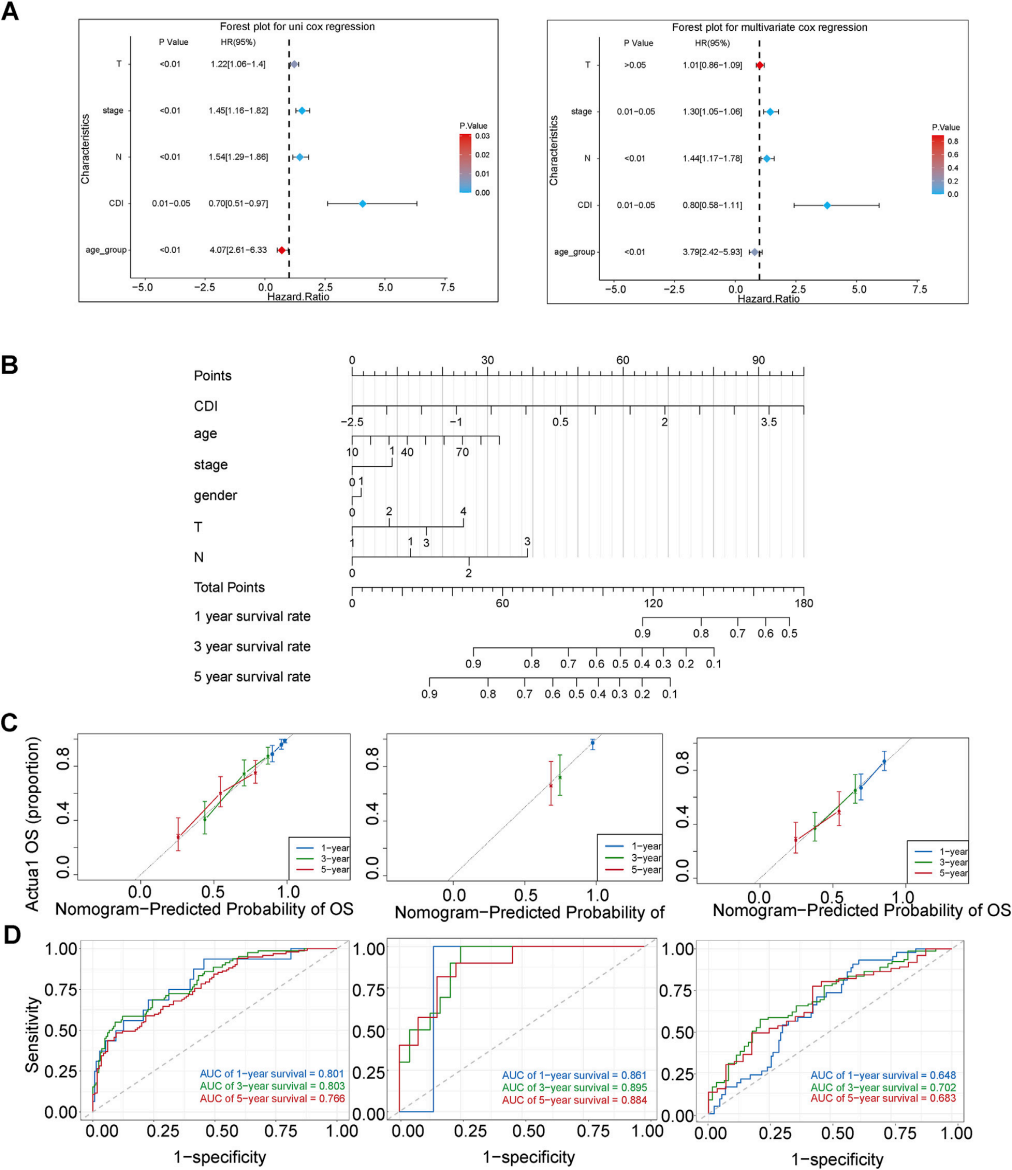
Establishment and assessment of the nomogram survival model. **(A)** Univariate analysis and multivariate analysis for the clinicopathologic characteristics and CDI in TCGA cohort. **(B)** A nomogram was established to predict the prognostic of malignant melanoma patients in TCGA cohort. **(C)** Receiver operator characteristic (ROC) analysis of nomogram in TCGA, GSE19234, and GSE65904 cohorts. **(D)** Calibration plots showing the probability of 1-, 3-, and 5-year overall survival in TCGA, GSE19234, and GSE65904 cohorts.

We performed univariate and multivariate Cox regression analyses to determine whether CDI could serve as an independent prognostic factor for melanoma. CDI (HR = 0.70, 95%CI: 0.51–0.97, p = 0.001) and stage (HR = 1.45, 95%CI: 1.16–1.82, p = 0.001) were considered as risk factors according to the univariate Cox regression analysis; they remained independent prognostic factors after multivariate analysis (Figure 8A). We developed a nomogram model including age, gender, stage, CDI, T stage, and N stage using multivariable Cox and stepwise regression analyses in the TCGA cohort to predict 1-, 3- and 5-year OS (Figure 8B). The ROC analysis results showed that the AUC values for 1-, 3- and 5-year survival predictions using the nomogram were at a high level, with values of 0.801, 0.803, and 0.766, respectively. In the validation set, the AUC ranged from 0.861 to 0.895 for GSE19234 and from 0.648 to 0.702 for GSE65904. (Figure 8D). Moreover, the calibration curves and AUC values obtained from ROC analysis in the three independent cohorts further supported the high accuracy of the nomogram in predicting the 1-, 3-, and 5-year survival of melanoma patients (Figures 8C, D).

**FIGURE 9:**
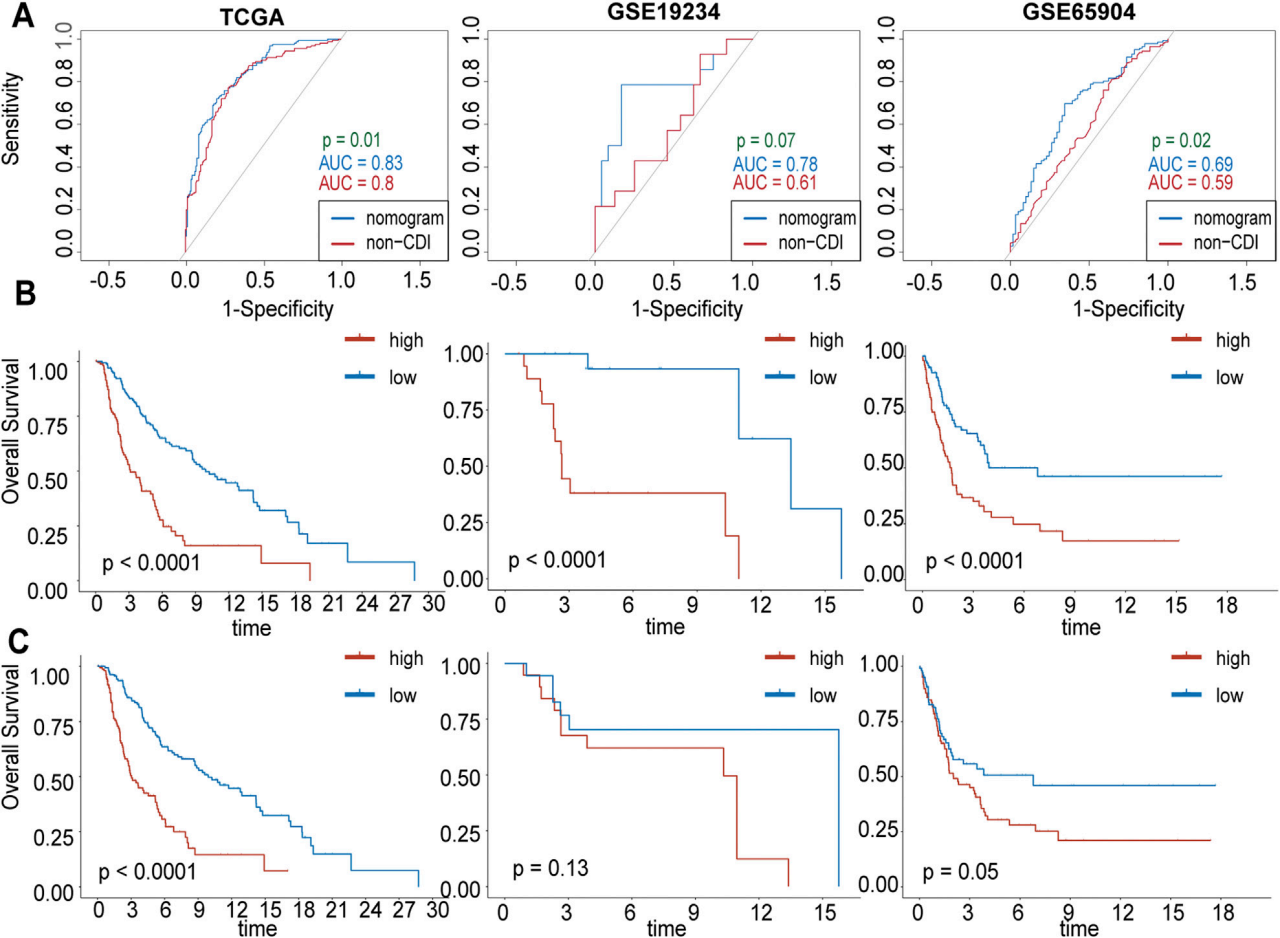
Comparison between CDI and without-CDI models. **(A)** Receiver operating characteristic (ROC) analysis of nomogram with and without CDI values in 3-year survival rate in TCGA, GSE19234, GSE65904 cohorts. **(B)** Kaplan–Meier survival curves grouped by nomogram scores. **(C)** Kaplan–Meier survival curves grouped by nomogram without CDI scores.

Additionally, we assessed the difference between the nomogram with and without CDI values by comparing their AUC values in predicting 3-year survival. Our results showed significant differences between the two models in TCGA-SKCM (p = 0.01) and GSE65904 (p = 0.02), indicating that the inclusion of CDI improved the accuracy of our prognostic predictions compared to existing clinical models (Figure 9A). Furthermore, the substantial disparities observed in the Kaplan–Meier curves, which were stratified based on individual scores, provide compelling evidence for the pivotal importance of CDI values in our model (Figures 9B, C).

## 4 Discussion

To the best of our knowledge, this study represents the first comprehensive analysis of 13 different modes of PCD in melanoma and establishes a cell death signature in the TCGA dataset, which was further validated in two external datasets (GSE19234 and GSE65904). We constructed a nomogram incorporating clinical features and the CDI which demonstrated excellent performance. Additionally, we investigated the correlation between CDI and immune regulators as well as drug sensitivity. The establishment and analysis of cell death models for melanoma can effectively stratify the prognosis of patients and provide new directions for further elucidating the mechanisms of tumor occurrence and development, as well as drug target research and development.

PCD involves intricate regulation and encompasses various mechanisms. Growing evidence suggests that it plays a crucial role in biological processes and has been implicated in the development and metastasis of malignant tumors for decades (Su et al., 2015). We created a feature consisting of ten genes (ATP6V0D1, CD74, GSTP1, MLKL, NFATC4, TRAF1, TRIM27, VPS13C, XBP1, and ZBP1) associated with PCD and found that it could predict overall survival in melanoma patients. ATP6V0D1, which encodes a subunit of the proton pump involved in endocytic pathways (Stevens and Forgac, 1997), was identified as a risk factor for melanoma survival. The STAT3 pathway, mediated by ATP6V0D1, consistently enhances alkaliptosis in tumor cells (Chen et al., 2023). CD74 is highly expressed on circulating tumor cells in the majority of melanomas, and its elevated expression in tumor cells may lead to increased levels of the sCD74 soluble form. Higher serum sCD74 levels are strongly correlated with prolonged survival in patients with advanced melanoma. (Loreth et al., 2021; Tanese and Ogata, 2024). Epigenetic silencing of GSTP1 is a common genetic alteration (>90%) in prostate cancer (Henrique and Jerónimo, 2004). Importantly, GSTP1 also plays a crucial role in melanoma by augmenting drug resistance and enhancing detoxification mechanisms, thereby positioning itself as a potential therapeutic target (Depeille et al., 2005). Our study found higher expression of GSTP1 in melanoma samples; elevated GSTP1 expression may be associated with poorer prognosis. MLKL, a key executor of necroptosis, regulates tumor development, progression, and metastasis through both RIPK3-dependent and independent mechanisms, including receptor internalization, extracellular vesicle formation, and inflammation regulation. Its upregulation in response to inflammation and tissue injury suggests that MLKL may play dual roles in cancer (Martens et al., 2021). NFATC4 is a transcription factor of the NFAT family, and its expression can effectively assess patient risk and prognosis, consistent with our study’s findings of high NFATC4 expression correlating with poorer prognosis. TRAF1 plays a pivotal role in pro-survival signal transduction downstream of TNFR superfamily members and has potential as a molecular therapeutic target for various human cancer types (Zhong et al., 2022). Our results suggest that TRAF1 is a prognostic factor in melanoma. As a member of the TRIM protein family, TRIM27 is a RING-mediated E3 ubiquitin ligase. TRIM27 may contribute to cell proliferation through the activation of p-Akt1, influence cell migration and invasion, and potentially serve as a predictor of distant metastasis in SNMM. It could also be considered a criterion for adjuvant chemotherapy following curative treatment (Kimura et al., 2023). Studies on the role of TRIM27 in cancer consistently report its carcinogenic effects (Yu et al., 2022), aligning with our research. Currently, research on the VPS13C gene remains limited; however, our study indicates that higher expression of VPS13C in tumor tissue is associated with improved prognosis, consistent with previous findings (Wu and Zhao, 2021). Our findings indicate that MLKL exhibits a clear anti-tumor effect in melanoma. XBP1 is a unique basic region leucine zipper (bZIP) transcription factor that not only promotes tumor cell proliferation but also participates in immune evasion, angiogenesis, hypoxia, invasion, and metastasis during tumorigenesis. Suppressing XBP1 expression can reduce tumor cell viability and drug resistance (Chen et al., 2020). We found that it is also a favorable prognostic factor in melanoma. ZBP1 is an interferon-induced cytosolic nucleic acid sensor that facilitates antiviral responses via RIPK3. We found higher expression of ZBP1 in tumor tissue, but patients with higher expression had better prognosis.

Tumor cells are able to survive due to the tumor microenvironment, which allows them to evade immune surveillance and drug interference (Zou et al., 2019). According to our analysis, the activity of various immune cells in the CDI low group was significantly higher than that in the high group, which includes a higher proportion of activated B cells, memory CD8^+^ cells, NK cells, and others. Activated B cells with high glycolytic and OXPHOS activity are found in melanoma patients and are associated with adverse reactions to immune checkpoint blockade therapy (Imahashi et al., 2022). Memory CD8^+^ T cells are capable of persisting and functioning in host tissues and tumors, mediating durable tumor immunity (Han et al., 2020) Natural killer cells are a specialized immune effector cell type whose activation is governed by the interaction of NK receptors with target cells, independent of antigen processing and presentation (Liu et al., 2021). Additionally, NK cells can exert anti-tumor responses without prior sensitization (Terrén et al., 2019). Immunosuppressive cells, such as MDSC and regulatory T cells, are more prominent in the low CDI group. Therefore, differences in immune infiltration may not necessarily reflect the strength of antitumor immunity. Further precise experimental validation is required to elucidate the relationship between programmed cell death (PCD) and the immune microenvironment in melanoma. Our study found that patients in the high CDI group had higher IC50 values for Axitinib, carmustine, docetaxel, temozolomide, paclitaxel, and dabrafenib. This indicates potential resistance to chemotherapy drugs used in melanoma treatment, leading to poorer prognosis in the high CDI group.

Furthermore, our analysis using the established CDI index revealed that PCD-related genes have a high predictive accuracy and robustness for patient survival in melanoma. The CDI low group showed relatively better survival outcomes. The 3-year survival AUC values were 0.668 for the TCGA cohort, 0.863 for GSE.19234, and 0.656 for GSE65904. After performing univariate and multivariate Cox regression analysis on important clinical variables, CDI was identified as an independent prognostic factor. We constructed a column plot incorporating CDI and important clinical indicators. The 3-year AUC value for the TCGA cohort was 0.803. Comparing the results with ROC analysis excluding the CDI value, our nomogram is more suitable for clinical practice.

Overall, our findings shed light on the association between PCD and melanoma prognosis and provide insights into potential therapeutic targets. The identification of the CDI and its correlation with immune modulation and drug sensitivity offer promising avenues for personalized treatment strategies in melanoma patients.

Melanoma is a highly immunogenic tumor characterized by numerous genetic mutations and represents a paradigmatic cancer for immunotherapy and targeted therapy research. Previous studies on melanoma and cell death have explored the impact of apoptosis-related pathways on melanoma progression and the influence of ferroptosis-related genes (TP53, CP, MAP1LC3A, and TF) on melanoma prognosis (Long and Pi, 2020; Chen et al., 2021; Liu Y. et al., 2022). In this study, we integrated the major 13 modes of cell death into a comprehensive CDI scoring system. Enrichment analysis, immune analysis, and drug sensitivity analysis were conducted based on CDI groups, and a clinically predictive model with good discriminative ability was developed. Further research can explore the regulatory mechanisms of gene mutations, immune infiltration, and identify more effective targets to improve patient survival and prognosis in melanoma.

In conclusion, this study establishes and validates a cell death gene signature for stratifying prognosis in melanoma patients, revealing the association between PCD and melanoma prognosis and providing insights into potential therapeutic targets. The CDI-based scoring model shows promising prognostic efficacy for melanoma patients.

## Data Availability

The original contributions presented in the study are included in the article/Supplementary Material; further inquiries can be directed to the corresponding authors.

## Supplementary material

SUPPLEMENTARY FIGURE 1 Variant landscape of programmed cell death genes in malignant melanoma patients. **(A, B)** KEGG and GO enrichment analyses based on DEGs. **(C, D)** Oncoplot of PCD-related genes in the TCGA cohort.

SUPPLEMENTARY FIGURE 2 KM survival analysis of each model gene in TCGA cohort. (blue: low expression group; red: high expression group).

SUPPLEMENTARY FIGURE 3 t-test of expression of each model gene between malignant melanoma tissues (red) and normal sample (blue). ****p < 0.0001; **p < 0.01; *p < 0.05; ns, “not significant”.
