# Supplementary figures and images for: Multiple cell-death patterns predict the prognosis and drug sensitivity of melanoma patients
Source: Front Pharmacol. 2024 Oct 8;15:1295687. doi: 10.3389/fphar.2024.1295687 (PMC11493598; doi:10.3389/fphar.2024.1295687)

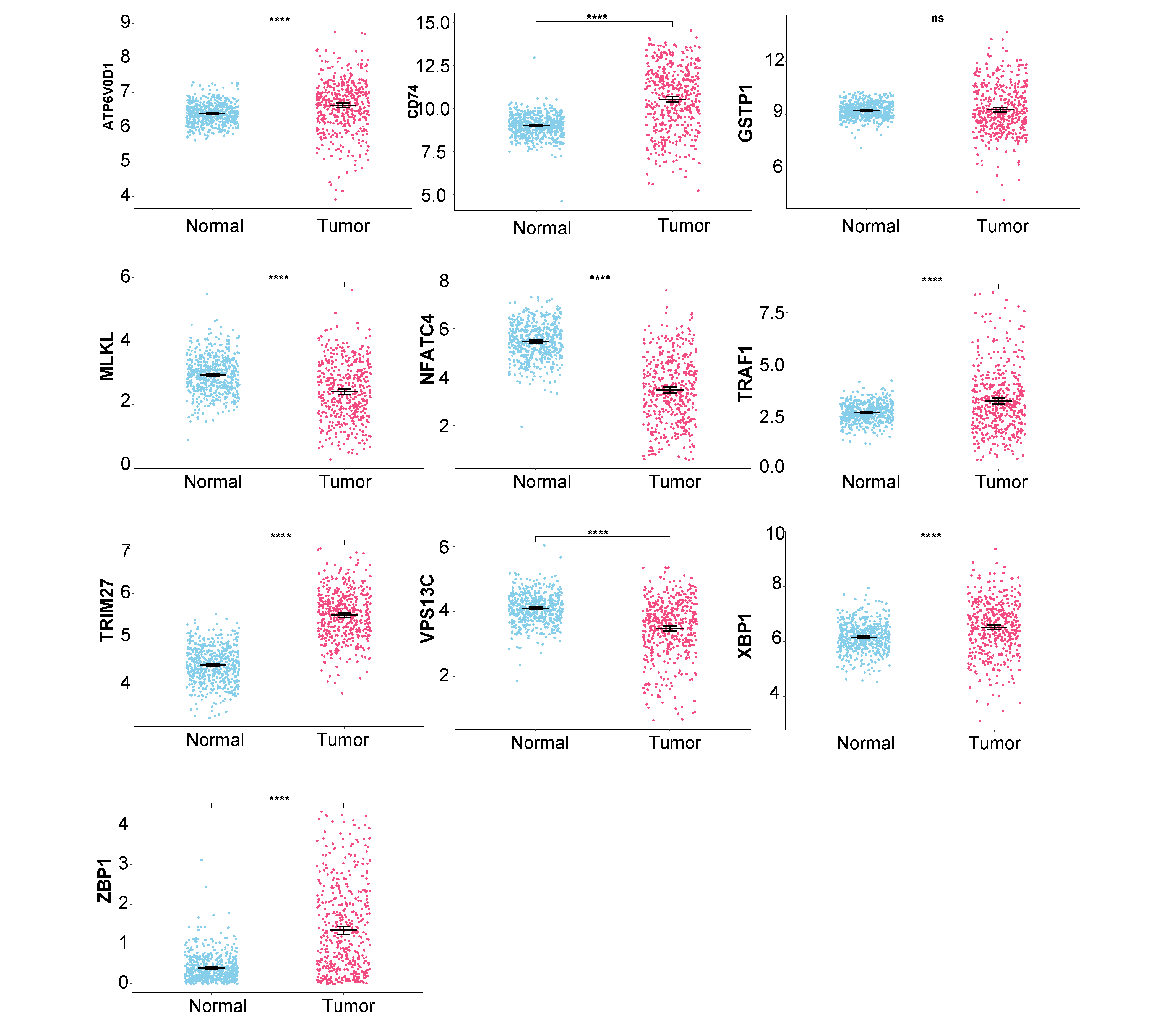

Supplement: Supplementary file 4 [file Image3.TIF]

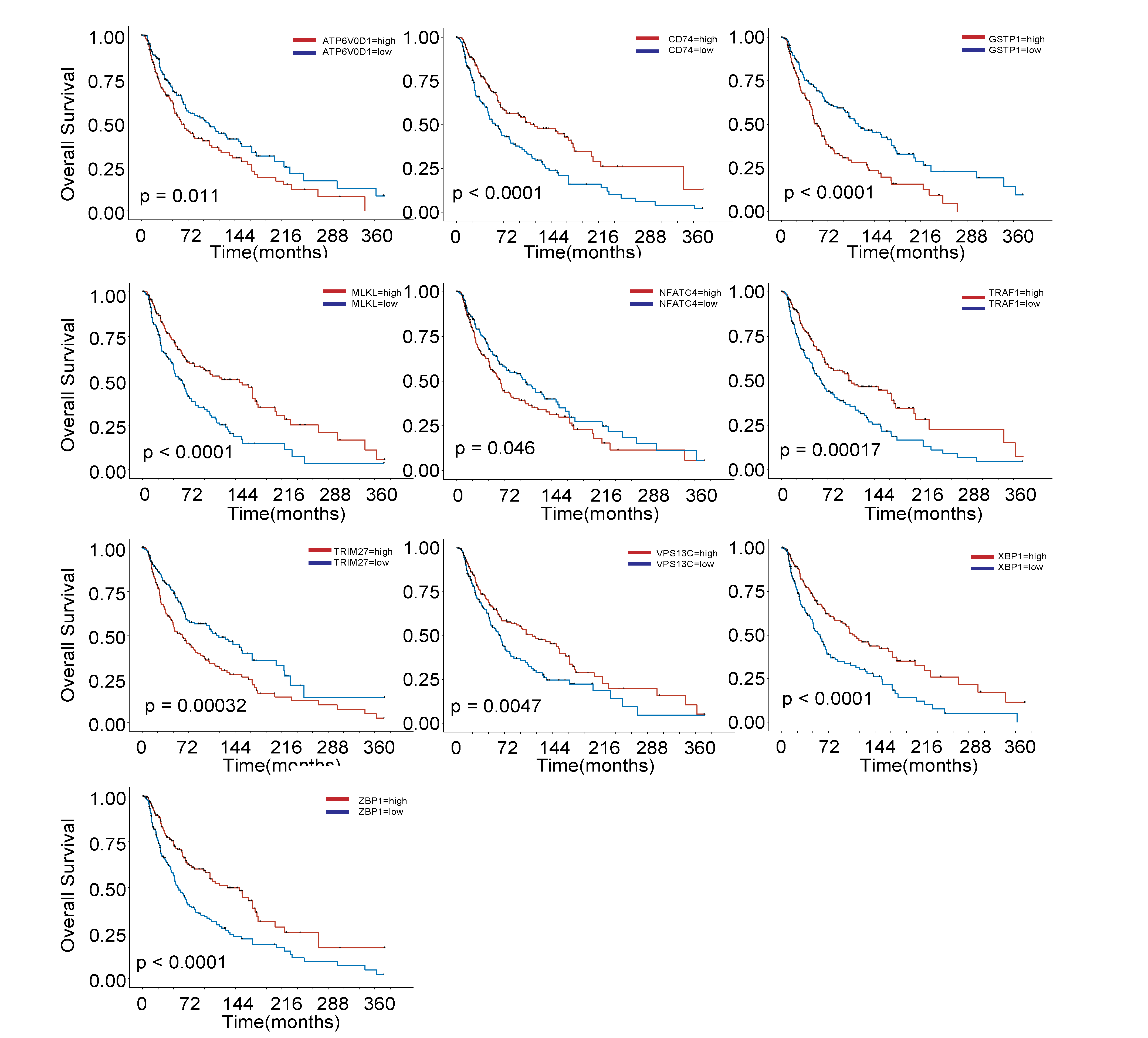

Supplement: Supplementary file 5 [file Image2.TIF]

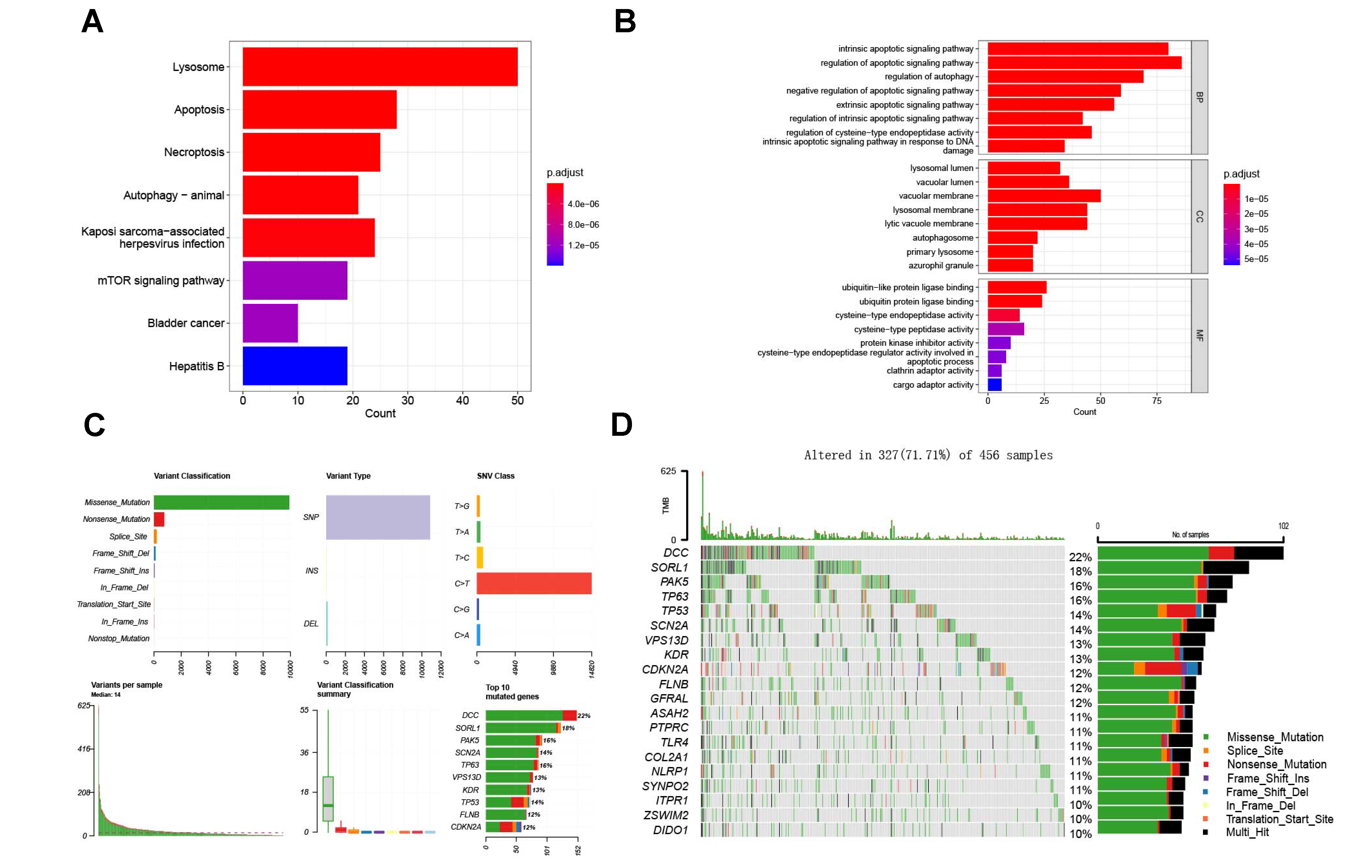

Supplement: Supplementary file 6 [file Image1.TIF]
